# Efficient light-driven hydrogen evolution and azo dye degradation over the GdVO_4_@g-C_3_N_4_ heterostructure†

**DOI:** 10.1039/d3ra02949b

**Published:** 2023-07-07

**Authors:** Fahad A. Alharthi, Adel El Marghany, Naaser A. Y. Abduh, Imran Hasan

**Affiliations:** a Department of Chemistry, College of Science, King Saud University Riyadh-11451 Saudi Arabia fharthi@ksu.edu.sa amarghany@ksu.edu.sa 439106262@student.ksu.edu.sa iabdulateef@ksu.edu.sa +966-507976713

## Abstract

A straightforward hydrothermal technique was used for the synthesis of a g-C_3_N_4_/GdVO_4_ (CN/GdV) heterostructure as an alternate material for energy and environmental applications. X-ray diffraction (XRD), scanning electron microscopy (SEM), transmission electron microscopy (TEM), and X-ray photoelectron spectroscopy (XPS) were used to characterize the synthesized g-C_3_N_4_ (CN), GdVO_4_ (GdV), and the CN/GdV heterostructure. The characterization results revealed the distribution of GdV over CN sheets. The as-fabricated materials were tested for their capacity to evolve hydrogen gas and degrade two azo dyes (Amaranth; AMR and Reactive Red2; RR2) in the presence of visible light. When compared to pure CN and GdV, the efficiency of CN/GdV toward hydrogen evolution was high, with H_2_ evolution of 8234, 10 838, and 16 234 μmol g^−1^ in 4 h, respectively. The CN/GdV heterostructure was able to degrade 96% and 93% of AMR (60 min) and RR2 (80 min), respectively. The enhanced activity with CN/GdV could be attributed to the type-II heterostructure and decreased recombination of charge carriers. The intermediate analysis of AMR and RR2 degradation was conducted using mass spectrometry (MS). The mechanism of photocatalysis was investigated and is discussed based on the optical and electrochemical characterizations. The efficient photocatalytic characteristics of CN/GdV could promote further research on metal vanadate nanocomposite materials.

## Abstract 2

A straightforward hydrothermal technique was used for the synthesis of a g-C_3_N_4_/GdVO_4_ (CN/GdV) heterostructure as an alternate material for energy and environmental applications.

## Graphical abstract

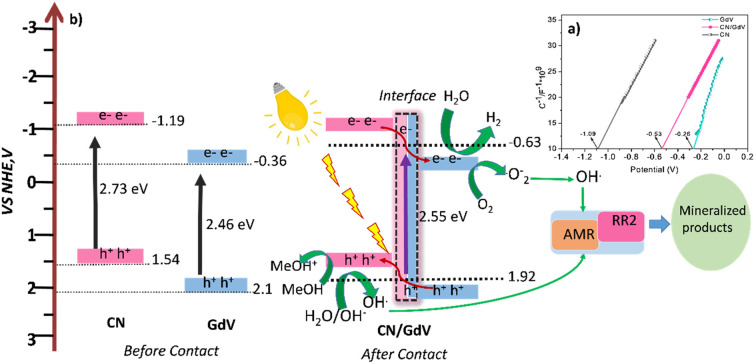

## 1. Introduction

Extensive industrialization and urbanization across the globe have been driven by the use of fossil fuels as the primary energy resources, but their unchecked use is also causing serious environmental issues while resources are rapidly depleting, both representing severe risks to society.^1,2^ Finding alternatives to fossil fuels is therefore of the utmost importance to help resolve the energy issues and reduce environmental pollution. To this purpose, researchers from all around the world are seeking to develop multifunctional materials that are renewable in nature.^3,4^ Hydrogen production through water splitting has become increasingly popular among alternative energy systems owing to its abundance of resources, light weight, enhanced energy density, environmentally friendly nature, and as H_2_ undergoes combustion with no or minimal pollution.^5,6^ Alongside this, the fabrication of visible-light-active catalysts is receiving a lot of attention as it can help hydrogen production and pollutant degradation through the conversion of solar energy into chemical energy. Monitoring the bandgap, surface area, wide light-absorption ability, the separation of photoinduced electron–hole pairs, stability, *etc.* are crucial factors in building visible-light-active catalysts.^7,8^

It is anticipated that clean and environmentally friendly hydrogen could soon replace fossil fuels to a large extent. Currently, H_2_ is created from natural gas at high temperatures using the steam reformation phenomenon, as well as employing noble metals, like Pt, Pd, and Ru.^9^ These techniques, however, typically have poor chemical kinetics, and are costly and have limited efficiency. Therefore, researchers are working on designing catalysts that are more economical, simple to manufacture, and free of precious metals.^10^ Another pressing global issue is environmental pollution. Many primary water bodies are polluted by the synthetic dyes used in the textile, culinary, tanning, and pharmaceutical industries.^11,12^ Numerous issues can occur as a result of the buildup of cancer-causing dyes in water, including the demise of aquatic life and reduced photosynthesis in plants.^13^ Amaranth (IUPAC: trisodium (4*E*)-3-oxo-4-[(4-sulfonato-1-naphthyl)hydrazono]naphthalene-2,7-disulfonat) and reactive red 2 (IUPAC: (5-[(4,6-dichloro-1,3,5-triazin-2-yl)amino]-4-hydroxy-3-(phenylazo)naphthalene-2,7-disulphonic acid) are two common azo dyes used in synthetic fibers, leather, paper, and phenol-formaldehyde resins. These highly stable azo dyes pollute water and upset the ecological balance and cause environmental issues. Therefore, an effective strategy for getting rid of them is in high demand. Other than photocatalysis, no other water purification technique, such as adsorption, membrane filtration, or coagulation, can achieve the total degradation of azo dyes from polluted water.^14–16^

Due to its ability to absorb visible light, good chemical stability throughout a broad pH range, and thermal stability, g-C_3_N_4_ (CN) is a metal-free polymeric semiconducting material with a bandgap of 2.5–2.8 eV that has gained much attention for a variety of photocatalytic applications.^17,18^ CN is commonly used as a replacement photocatalyst to degrade organic pollutants, reduce CO_2_, and catalyze chemical reactions under visible light because of its advantageous band position.^19,20^ However, the band gap of bulk CN is fairly broad for the absorption of visible light, which is defined as light with a wavelength shorter than 450 nm. Fast charge-carrier recombination is produced by the conjugated polymeric structure of CN as well as the stacking between the aromatic layers and the decreased specific surface area. Thus, it is highly desirable to create a unique CN-based photocatalyst with a smaller band gap, better charge separation and migration, and a large specific surface area.^21,22^

Researchers have focused much effort on nanostructured rare earth metal orthovanadates (AVO_4_; A = Ce, Fe, Sm, Eu, Sm, and Gd) because of their outstanding features. Generally speaking, AVO_4_ has two types of polymorphism: tetragonal zircon and monoclinic monazite. GdVO_4_ (GdV) is a visible-light-active photocatalyst material with a narrowband gap (2.1–2.5 eV) and fluorescence capabilities, which has led to it receiving the most attention in this regard and hence it is widely used in the fields of laser technology and optoelectronic devices.^22,23^ Additionally, it has been demonstrated that GdV has potential use in environmental applications. It has been exploited as a visible-light-active photocatalytic material for dye degradation.^24–27^ However, the photocatalytic activity of GdV is still poor, and so further structural modification with other carbon-based materials, metal oxides, or any other semiconductor is highly appreciated.^28^ At the same time, due to its low quantum efficiency, pure g-C_3_N_4_ has a hard time achieving its full photocatalytic potential. Elemental doping, nanosheet reduction, and heterojunction production are just a few of the methods that have been tried and tested to increase g-C_3_N_4_'s quantum efficiency. In addition, the light absorption and photocatalytic activity of nanosheets can be enhanced through the exposure of the interior atoms. The combination of two narrow bandgap semiconductors with different band positions results in the generation of heterostructures. Three different types of heterojunctions—straddling gap (type-I), staggered gap (type-II), and broken gap—can develop when two different types of semiconductors with different energy band structures (type-III) are combined.^29,30^ Under light stimulation, however, only the type-II heterojunction is capable of achieving efficient carrier interface transfer and spatial separation.^31^ GdVO_4_ nanowires have been used as a catalyst for hydrogen generation through methanol splitting, with an observed hydrogen evolution of 42 μmol h^−1^.^32^ GdVO_4_/g-C_3_N_4_ was synthesized by an ultrasonic dispersion method and used for the photocatalytic degradation of tetracycline hydrochloride and managed to degrade 91% in 3 h.^33^ In another report, a milling and heating synthetic approach was followed to fabricate a GdVO_4_/g-C_3_N_4_ nanocomposite, which was used for the degradation of Rhodamine B under visible light.^34^ Even though the hydrothermal technique appears straightforward and has numerous benefits, it has not been investigated for the construction of g-C_3_N_4_/GdVO_4_ (CN/GdV) heterostructures.

In the present work, the hydrothermal method was used for the efficient fabrication of the g-C_3_N_4_/GdVO_4_ heterostructure. The obtained CN/GdV heterostructure was used for photocatalytic hydrogen evolution and the degradation of Amaranth and Reactive red 2 dyes. Enhanced light-driven activity was observed in the CN/GdV heterostructure when compared to pristine GdV and CN. Based on the bandgap, Mott–Schottky, and LC-MS results, a detailed mechanism of the photocatalysis is discussed.

## 2. Experimental

### 2.1 Materials

Melamine, Gd (NO_3_)_3_·6H_2_O, NH_4_VO_3_, methanol, ethylene diamine tetra acetic acid (EDTA), tertiary butyl alcohol (TBA), benzoquinone (BQ), and benzoquinone were purchased from Fisher Scientific Ltd (Waltham, MA, USA) and used without further treatment. Double-distilled (DI) water was used completely throughout the experiment for washings and for the preparation of the solutions.

### 2.2 Synthesis of CN, GdV, and the CN/GdV heterostructure

**Scheme 1:**
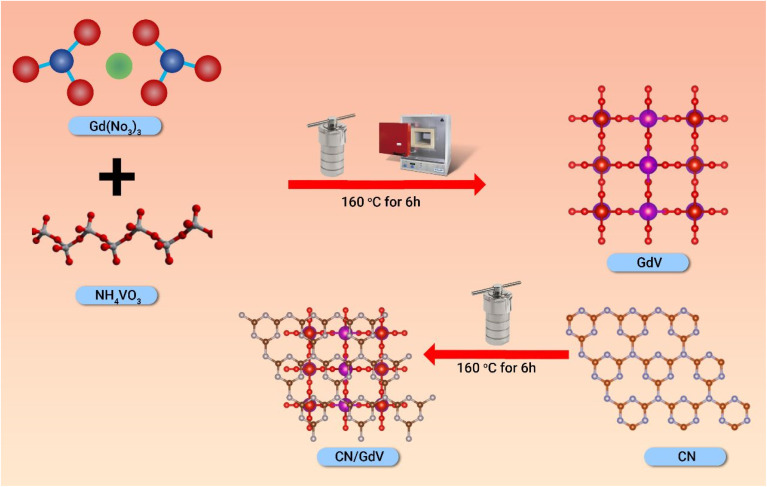
Synthesis of the CN/GdV heterostructure.

In this work, the heterostructure and pristine materials were synthesized in three main steps:

Step 1, CN was synthesized using melamine as the precursor, and then the melamine was heated to 600 °C in a muffle furnace for 6 h to undergo a thermal polycondensation. Later, the crucible was taken out of the furnace and ground in an agate mortar once it had cooled to room temperature. Step 2 entailed preparing the GdV *via* a straightforward hydrothermal process, with 0.2 g of Gd (NO_3_)_3_ ·6H_2_O and 0.4 g of NH_4_VO_3_ dissolved in 50 mL of double-distilled water and sonicated for 30 min to achieve a uniform mixture. The mixture was then placed in an autoclave lined with Teflon and heated to 160 °C for 6 h. The collected precipitate was then dried at 60 °C for 24 h after being washed three times with water and ethanol. Finally, in step 3, the CN/GdV heterostructure was fabricated by mixing 1 : 1 (10 mg) GdV and CV nanosheets in 25 mL of water and heating the mixture hydrothermally at 160 °C for 6 h. In addition to the above, the same washing and drying procedures were also followed. The samples were stored carefully before using them for the later photocatalytic applications. The synthetic procedure is schematically given in Scheme 1.

### 2.3 Photocatalytic hydrogen evolution

A precise amount (25 mg) of CN, GdV, and CN/GdV was added to 50 mL of water in a quartz round-bottom flask. Next, 20 mL of methanol was added to the solution, and then nitrogen gas was used to cleanse the container. A 300 W Xe bulb (light intensity = 85 mW cm^−2^, with filter *λ* > 400 nm) was utilized as a visible-light source and applied while the solution was stirred continuously using a magnetic stirrer. A gas chromatograph with a thermal conductivity detector was used to collect and measure the evolved gas.

### 2.4 Dye-degradation studies

Degradation studies were carried out using the as-synthesized materials by selecting Amaranth (AMR) and Reactive Red2 (RR2) as model pollutants. A Xe lamp of 300 W (light intensity = 85 mW cm^−2^, with filter *λ* > 400 nm) was used as the visible-light source. A standard solution containing 100 mg of L^−1^ dyes was prepared and diluted as needed. In a round-bottom flask, the dyes were added, kept at the optimized pH and catalyst dosage, and then subjected to sonication. In order to achieve adsorption/desorption equilibrium, the solution was later constantly agitated. A 3 mL aliquot was taken at an interval of 10 min, centrifuged, and the dye solution's absorbance was measured by UV-visible spectrometry at the appropriate wavelength. The deterioration% was calculated using eqn (1) below.1
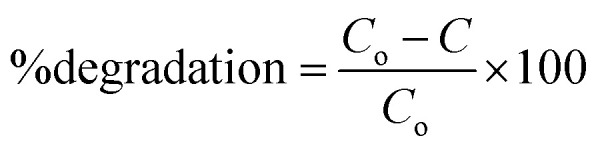
where *C*_o_ is the initial and *C* is the final concentration of the dyes, respectively.

### 2.5 Characterization

A Bruker D2 Phaser XRD system was used to record the X-ray diffraction (XRD) patterns of the materials. JEOL JSM 840A and JEOL/JEM 2100 instruments were used for the SEM and TEM analyses, respectively. X-Ray photoelectron spectroscopy (XPS) was performed on an omicron spectrometer. Photoluminescence studies were performed using 5 mg L^−1^ of the photocatalysts in a RF-6000 spectrofluorometer. Absorbance of dye solution was measured using a Shimadzu 1600 model. A PerkinElmer ARNL 580C system was used to measure hydrogen evolution. The electrochemical performance was studied using a CHI660E setup consisting of working (glassy carbon with a 1.80 mm^2^ geometric surface area), reference (Ag/AgCl), and counter (Pt wire) electrodes. The glassy carbon electrode was polished well with alumina kit and then subjected to sonication in ethanol and dried. Next, 5.0 μL of the photocatalyst was drop-cast over the glassy carbon electrode and then dried in the oven and finally used to record the current response and Mott–Schottky plots in the presence of visible light.

### 2.6 LC-MS analysis of dye degradation

The degradation products of amaranth (AMR) and reactive red 2 (RR2) were examined using a PerkinElmer LC-MS PE Sciex API/65 spectrophotometer. A 75/25 (v/v) acetonitrile–water mixture was used as the mobile phase and filtered using a 0.22 μm Millipore syringe filter, with 20 μL the injection volume and an elution flow rate of 0.8 mL min^−1^. The chromatographic column eluent was made to pass through the UV-visible diode array detector and the mass analyzer. The mass spectrometer analysis was carried out in the positive ions mode in the mass range of 0 to 500 *m*/*z*.

## 3. Result and discussion

**Fig. 1:**
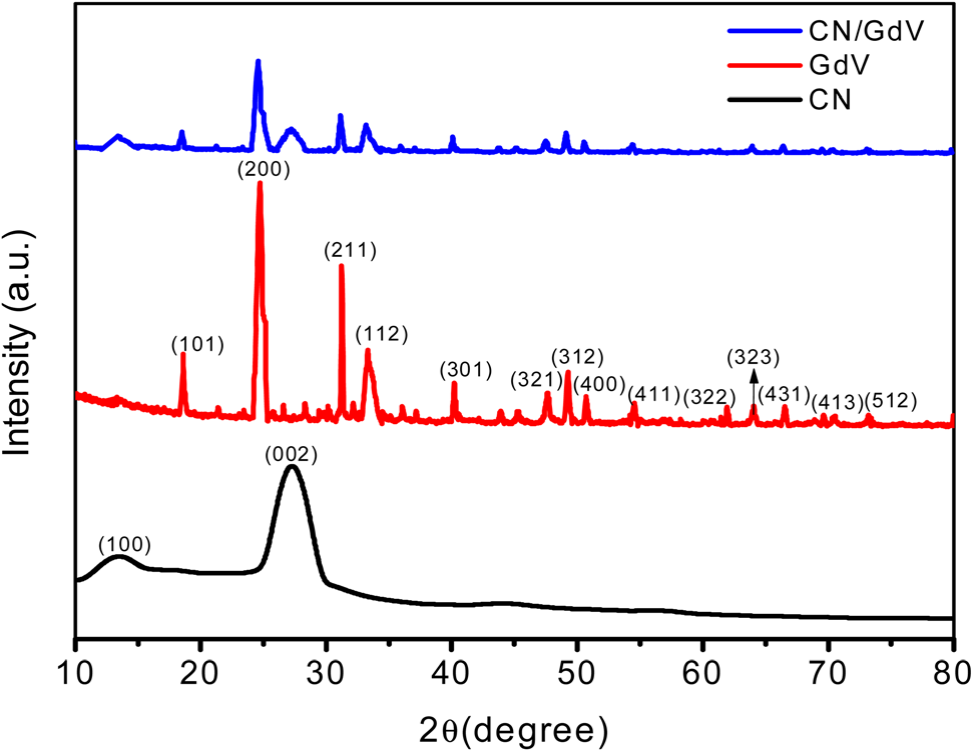
X-ray diffraction patterns of CN, GdV, and the CN/GdV heterostructure.

XRD studies were used to examine the crystallinity and orientation of the prepared samples. The XRD pattern of CN is depicted in Fig. 1, exhibiting distinctive peaks at 27.2° and 13.4°, which stand for the (002) and (100) planes, respectively and also corresponded to the JCPDS# 87-1526. Pristine GdV exhibited peaks at 2*θ* 18.69°, 24.73°, 31.22°, 33.32°, 40.21°, 47.72°, 49.30°, 50.71°, 54.48°, 61.96°, 64.12°, 66.60°, 69.91°, and 73.32° corresponding to the (101), (200), (211), (112), (301), (321), (312), (400), (411), (322), (323), (431), (413), and (512) crystallographic planes, which was is in good agreement with reference data JCPDS# 16-0452.^35^ The X-ray diffraction patterns proved that GdV was a single-phase material devoid of any impurities like gadolinium and vanadium oxides. In contrast, CN/GdV contained both CN and GdV diffractions. The (002) peak of the heterostructure was shifted to a lower angle, which was in line with findings for other CN composite materials reported in the literature. The small change in the peak position may be due to an overlap of the crystal planes. The peak shift was a consequence of the size distinction between C and Gd ions, which accounted for the observed change. Additionally, the heterostructure had a slightly wider peak and lower peak intensity. Furthermore, the addition of Gd did not alter the crystal structure of CN. These findings prove that the CN host was successfully integrated with GdV.

**Fig. 2:**
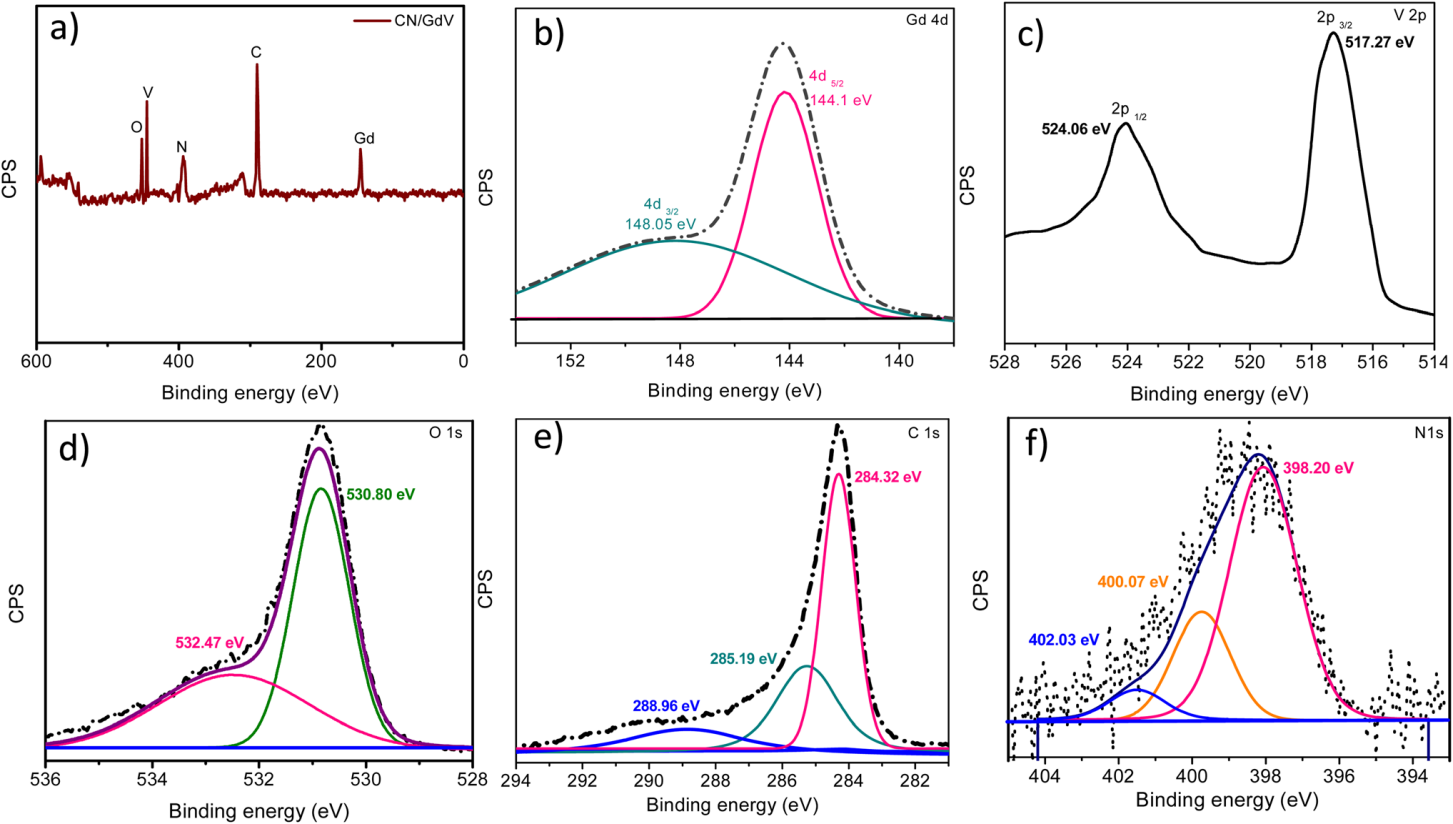
XPS of the CN/GdV heterostructure: (a) survey spectra of CN/GdV, (b) Gd 4d, (c) V 2p, (d) O 1s, (e) C 1s, and (f) N 1s.

With the help of XPS analysis (Fig. 2), we investigated the elemental makeup of the CN/GdV heterostructure. Fig. 2a depicts the survey spectrum of CN/GdV, which indicated the presence of Gd, V, O, C, and N atoms in the heterostructure. The high-resolution XPS spectra of Gd 4d (Fig. 2b) consisted of two peaks that may be attributed to the Gd 4d_5/2_ and Gd 4d_3/2_ energy states, respectively. Spin–orbit coupling was responsible for the Gd 4d_5/2_ core-level splitting, while the 4d coupling with 4f valence band electrons was responsible for the dominant spectral features.^36^ Two distinct peaks at binding energies of 517.27 and 524.06 eV, corresponding to the V 2p_3/2_ and V 2p_1/2_ electronic states of V 2p, could be seen in the deconvoluted spectra of V 2p (Fig. 2c). In addition, V^5+^ and V^4+^ were identified as vanadium's oxidation states.^37^Fig. 2d displays the XPS spectra of O 1s, which exhibited deconvoluted peaks at 530.20, and 532.47 eV, attributed to C–O, and O–C=O, respectively. The binding energies at 284.32, 285.19, 288.96 eV in the high-resolution spectra of C 1s (Fig. 2e) were consistent with the presence of sp^2^-bonded carbon and N–C=N. Three fitted peaks at 398.20, 400.07, and 402.03 eV were found after deconvoluting the N 1s peak (Fig. 2f). These corresponded to the pyridine N, the pyrrolic N, and the graphitic N, respectively.

**Fig. 3:**
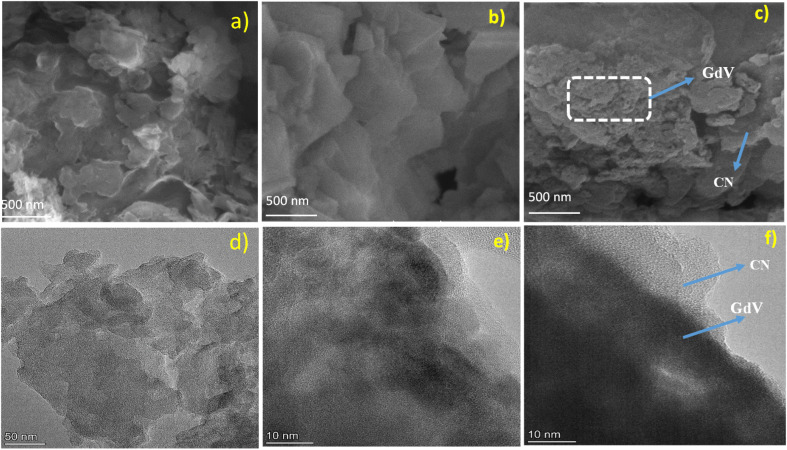
SEM and TEM monographs of (a), (d) CN, (b), (e) GdV, (c) and (f) the CN/GdV heterostructure.

SEM was used to examine the surface morphologies of CN, GdV, and CN/GdV. In the scanning electron micrograph of CN, a wrinkly sheet-like structure was easily discernible (Fig. 3a). The irregular pellet-like structure of GdV, shown in Fig. 3b, was not uniform in size and was found randomly throughout the material. In Fig. 3c, we see the FESEM image of CN/GdV, which shows that the GdV pellets were closely packed and randomly dispersed across the CN sheets. Alternatively, sheets of CN covered the GdV pellets in a thick layer. The SEM images show that CN/GdV had been formed successfully; this heterostructure's photocatalytic property would benefit from the combination of GdV and CN, which were both shown to be present in the images. Next, transmission electron microscopy (TEM) analysis was conducted to learn more about the morphology of the synthesized material. Fig. 3d and e present the micrographs of CN and GdV, respectively, which agree very well with what was seen under the SEM. The TEM image of CN/GdV (Fig. 3f) confirmed the pellet-like structure of the GdV and its random distribution across the CN sheets. The layer of CN sheets neatly overlapped the GdV pellet. The HR-TEM image (Fig. S1†) revealed a close interface was formed between the two phases of CN and GdV. Consistent with the XRD, the detected lattice fringes of *d* = 0.328 nm and *d* = 0.306 nm corresponded to the (211) crystallographic planes of GdV and (002) crystallographic plane of CN, respectively.

**Fig. 4:**
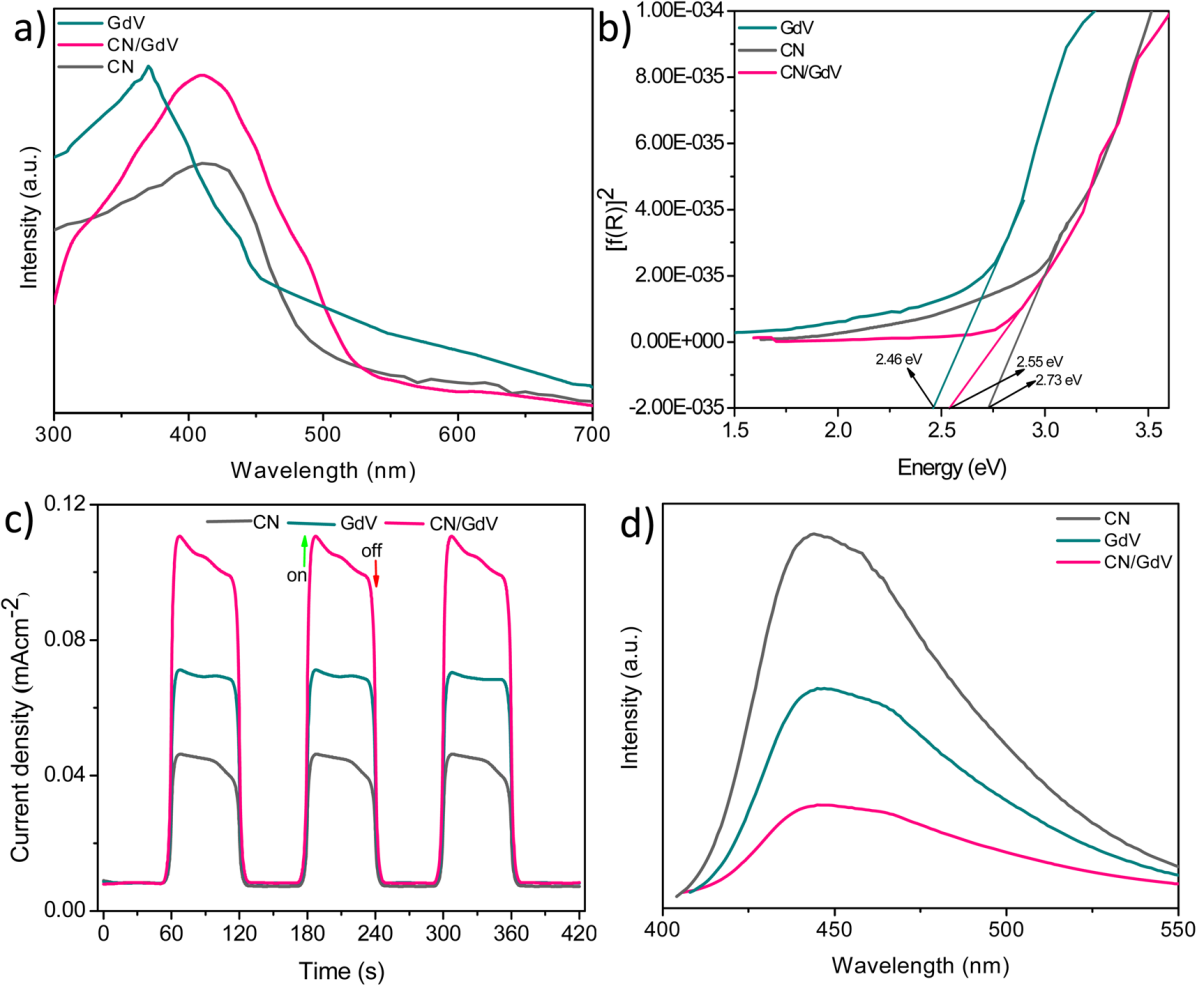
(a) UV-DRS spectra, (b) Kubelka–Munk curves, (c) transient photocurrent response, (d) photoluminescence spectra.

Insights from the optical and photoelectrochemical properties of materials play an important role in their photocatalytic applications. The optical properties of CN, GdV, and CN/GdV were examined using UV-diffusion reflectance spectroscopy (DRS) and the spectra are depicted in Fig. 4a. CN exhibited its maximum light absorption at around 450 nm with an absorption edge up to around 520 nm. The absorption edge of GdV was found at around 455 nm with a maximum absorption at 390 nm. Upon fabricating the CN/GdV heterostructure, a red-shift in absorption was observed compared to the absorption of GdV, indicating the ability of the heterostructure to absorb visible radiation effectively. The corresponding Kubelka–Munk factor for finding the bandgap of the materials was evaluated, as shown in Fig. 4b. The bandgaps of CN, GdV, and the CN/GdV heterostructure were found to be 2.73, 2.46, and 2.55 eV, respectively. The formation of a heterostructure between CN and GdV clearly showed the bandgap tuning and caused enhanced photocatalytic activity. Charge separation in the synthesized materials was examined using transient photocurrent measurements using an electrochemical workstation. Fig. 4c indicates the ability to produce current in all three synthesized materials in the presence of light. The current responses in GdV and the CN/GdV heterostructure were 1.5 and 2.5 times higher than in CN. The enhanced current response in CN/GdV showed the efficient charge separation during the photoredox reaction. Photoluminescence studies were performed to further understand the electron–hole separation. In Fig. 4d, the photoluminescence intensity of CN was higher than for GdV and CN/GdV. The decreased intensity in the CN/GdV heterostructure further indicated the efficient charge separation and hence this would be expected to reduce the recombination of charge carriers. These optical and photoelectrochemical results indicated the enhanced photocatalytic performance in CN/GdV due to the formation of the heterostructure.

### 3.1 Photochemical hydrogen evolution

**Fig. 5:**
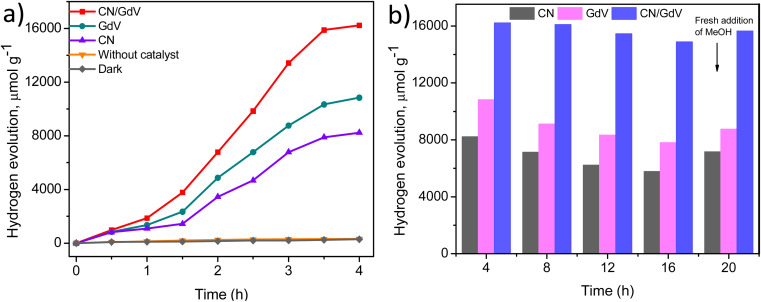
(a) H_2_ evolution graph and (b) bar chart for CN, GdV, and the CN/GdV heterostructure.

The optical and electrical properties of CN, GdV, and the CN/GdV heterostructure suggested a likelihood that they will absorb visible light and demonstrated their capacity for light-driven redox processes. As a result, the synthesized materials' potential for light-driven hydrogen evolution in the presence of a sacrificial agent (methanol) was evaluated. Fig. 5a illustrates the rate of H_2_ evolution for various materials over a period of 4 h and suggested that H_2_ evolution only takes place in the presence of light. It was found that a greater amount of hydrogen was evolved by GdV (10 838 μmol) than CN (8234 μmol). Also, 16 234 μmol of H_2_ evolution was observed for the CN/GdV heterostructure. The efficiency in CN/GdV was almost doubled compared to pristine CN and GdV. The formation of a heterostructure through the interface between CN and GdV could decrease the recombination of charge carriers, which could be the reason for the enhanced activity in CN/GdV. One of the crucial elements of a photocatalyst's practical application is its stability. Studies on the stability of CN, GdV, and the CN/GdV heterostructure were conducted under ideal circumstances for various cycles (each run constituted 4 h). Fig. 5b shows that the stability of CN/GdV remained high even up to 4 cycles when compared to the stability of pristine CN and GdV. The amount of hydrogen evolution was found to increase (at the 5th cycle) after the addition of fresh methanol (after the 4th cycle), which suggested that the reduced methanol concentration may have contributed to the drop in hydrogen evolution. The apparent quantum yield (AQY) was examined and found to be 4.82% (Fig. S2†). These results indicate the stability as well as efficiency improvement upon combining CN and GdV materials. The CN/GdV showed enhanced activity over many reported methods, as observed in Table 1.

**Table 1:** Comparison of the CN/GdV heterostructure's H_2_ evolution over other reported methods in the literature

S. no.	Material	Sacrificial agent	Light source	H_2_ evolution	Ref.
1	SmV/S–C_3_N_4_	TEOA	400 W Xe light	22 618 μmol g^−1^	21
2	Nb_2_O_5_/g-C_3_N_4_	TEOA/Pt	300 W Xenon lamp/(*λ* > 400 nm)	50.65% and 14.75% at 405 nm and 420 nm	38
3	ZnIn_2_S_4_/g-C_3_N_4_	TEOA	300 W Xenon lamp/(*λ* > 420 nm)	7.05% at 420 nm	39
4	LaVO_4_/CN	10 mL of FFA or TEOA	The Xenon lamp (300 W, 250 mW cm^−2^) stimulated sunlight	0.95 mmol g^−1^	40
5	g-C_3_N_4_/ZnIn_2_S_4_	10% lactic acid	300 W Xe lamp, *λ* > 420 nm	10.92 mmol h^−1^ g^−1^	41
6	AgPd/2D g-C_3_N_4_	Formic acid/sodium format	300 W Xenon lamp/(*λ* > 400 nm)	231.6 mmol h^−1^	42
**7**	**CN/GdV**	**Methanol**	**300 W Xe lamp light intensity** = **85 mW cm^−^**^**2**^**, with filter *λ*** > **400 nm**	**16 234 μmol g^−1^**	**Present work**

### 3.2 Photocatalytic degradation of dyes

**Fig. 6:**
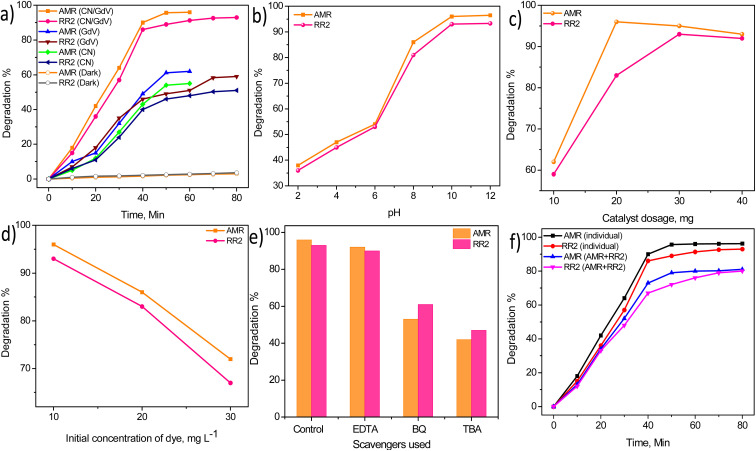
(a) Degradation of AMR and RR2 under different conditions, (b) effect of pH, (c) effect of the amount of the catalyst, (d) effect of the initial concentration. (e) Scavenger studies. (f) Simultaneous degradation of AMR and RR2.

The properties of the as-synthesized materials to absorb light was further evaluated by evaluating the degradation of commonly used azo dyes amaranth (AMR) and reactive red 2 (RR2). Fig. 6a shows the degradation profile of AMR and RR2 in the presence of visible light and different catalysts, indicating the degradation of the dyes did not occur under dark conditions. CN with its visible active characteristic managed to degrade just 55% and 51% of AMR (60 min) and RR2 (80 min), respectively. Pristine GdV was found to be more active and showed slightly higher degradations of AMR (62%) and RR2 (59%). With the CN/GdV heterostructure, enhanced activity was observed and the degradations of AMR and RR2 were found be 96% and 93%, respectively. The superior light-driven degradation of dyes observed in CN/GdV could probably be attributed to the formation of a heterostructure through the interface. This interface and heterojunction led to the decreased recombination of photoexcited electrons and holes and resulted in the enhanced photocatalytic activity. The ability of CN/GdV toward the degradation of dyes was further subjected to the optimization of different parameters that could affect the degradation. The pH of the reaction medium is one of the important feature of photocatalysis that needs optimization. The pH of the solution was varied from 2 to 12 and the results are shown in Fig. 6b. The results indicated that the degradations of both AMR and RR2 were low under acidic conditions, and were found to increase in basic media. The maximum degradation of both the dyes was observed at pH 10 and remained almost the same even at pH 12. Hence pH 10 was considered optimum and used for the further studies. The amount of the catalyst (CN/GdV) toward the degradation of AMR and RR2 was evaluated and the results are shown in Fig. 6c. It was found that 20 mg for AMR and 30 mg for RR2 were optimum. When the amount of catalyst added was more than optimum, the efficiency decreased slightly due to the loss of its surface property and the formation of a non-transparent system during photocatalysis. The initial concentration of the dyes subjected to degradation was varied and the ability of CN/GdV toward their breakdown was examined. It could be found from Fig. 6d that 10 mg L^−1^ of both the dyes showed efficient degradation in the presence of CN/GdV, but when upon increasing the concentration of the dyes, a decrease in the degradation percentage was observed. The reactive oxygen species (ROS) generated during the light-driven reaction in the presence of a catalyst can be found through scavenger studies. Different scavengers for holes (EDTA), hydroxyl ions (TBA), and super oxide radicals (BQ) were studied here (Fig. 6e). It was found that super oxide radicals and hydroxyl ions were the major active species generated during photocatalysis. Further, both dyes were mixed in a ratio of 1 : 1 and the absorbance of AMR and RR2 was measured individually in their respective wavelengths and the results are given in Fig. 6f. The CN/GdV heterostructure showed efficiency toward the simultaneous degradation of the dyes, with a slight deviation when compared to their individual degradation. The degradations of AMR and RR2 in the mixture were found to be 81% and 80%, respectively. The above result indicated the profound light-driven activity of the CN/GdV heterostructure for probable practical applications.

### 3.3 Analysis of the degradation intermediates using mass spectroscopy

**Scheme 2:**
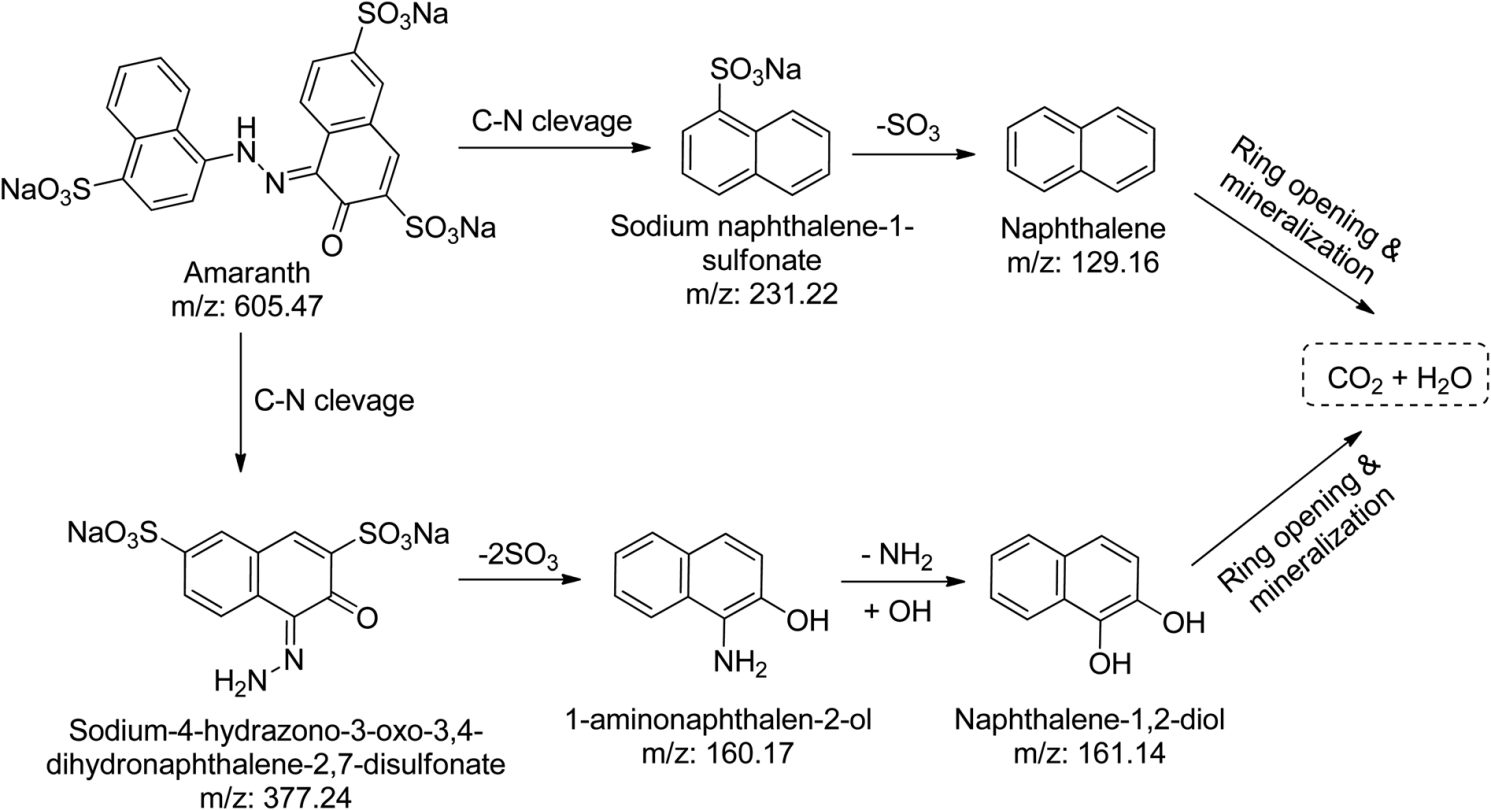
Proposed degradation pathway of amaranth dye in the presence of CN/GdV.

**Fig. 7:**
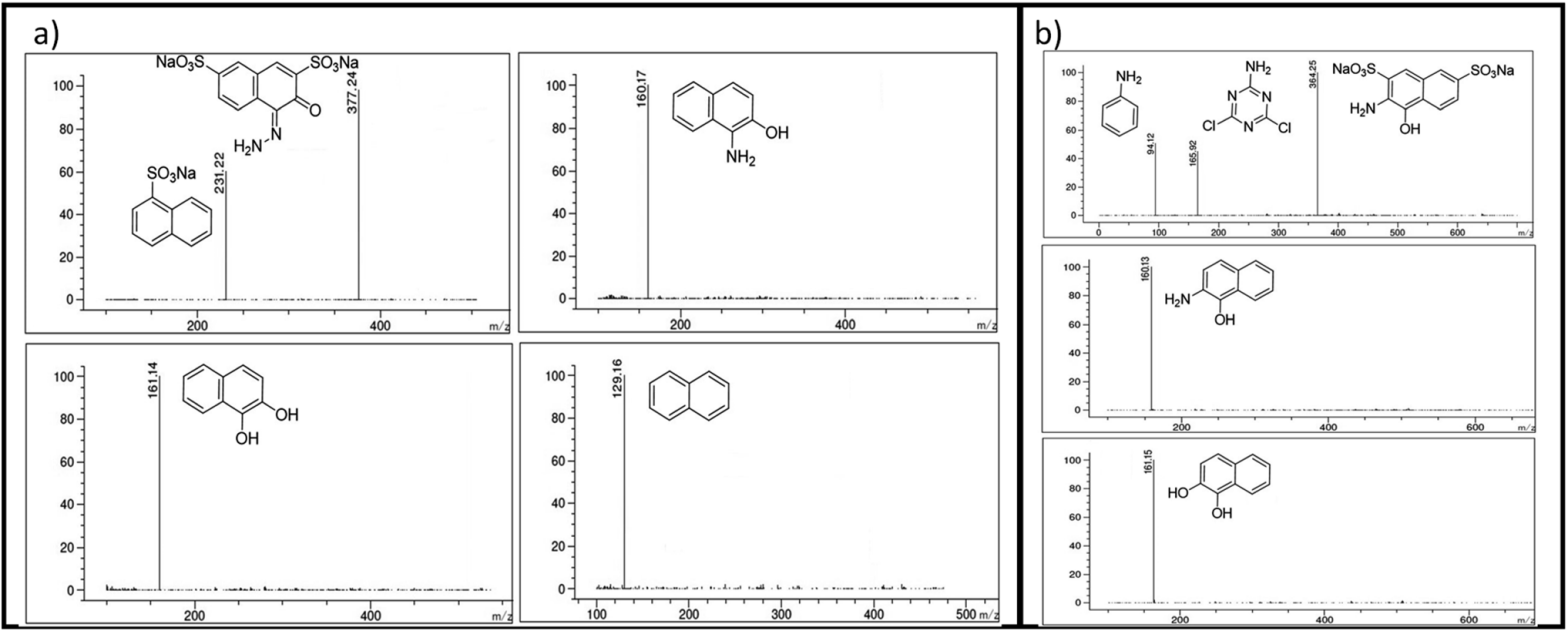
Mass spectra of the intermediates of (a) AMR and (b) RR2.

Using the CN/GdV heterostructure, the photocatalytic degradation of two common azo dyes, here amaranth and reactive red 2, was investigated under visible light. The proposed photocatalytic breakdown mechanism for amaranth dye is shown in Scheme 2. By analyzing the *m*/*z* peaks of the reaction mixtures during the irradiation, the dye byproducts produced during the irradiation were identified by LC-MS (Fig. 7a). The photocatalytic degradation reaction pathways were studied in light of ROS attack on the AMR and RR2 structures. Beginning with sodium naphthalene-1-sulfonate (*m*/*z*: 231.22) and sodium-4-hydrazono-3-oxo-3,4-dihydro naphthalene-2,7-disulfonate (*m*/*z*: 377.24), which may be attacked by radicals, the C–N cleavage of amaranth results in the creation of these two intermediates. In the next steps, sodium naphthalene-1-sulfonate loses a SO_3_ group to produce naphthalene (*m*/*z*: 129.16), while sodium-4-hydrazono-3-oxo-3,4-dihydro naphthalene-2,7-disulfonate loses two SO_3_ groups to produce 1-aminonaphthalen-2-ol (*m*/*z*: 160.17), and the deamination of 1-aminonaphthalen-2-ol produces naphthalene-1,2-diol (*m*/*z*: 161.14). Finally, the mineralization of naphthalene and naphthalene-1,2-diol into small molecules may result from the ring-rupture mechanism.

**Scheme 3:**
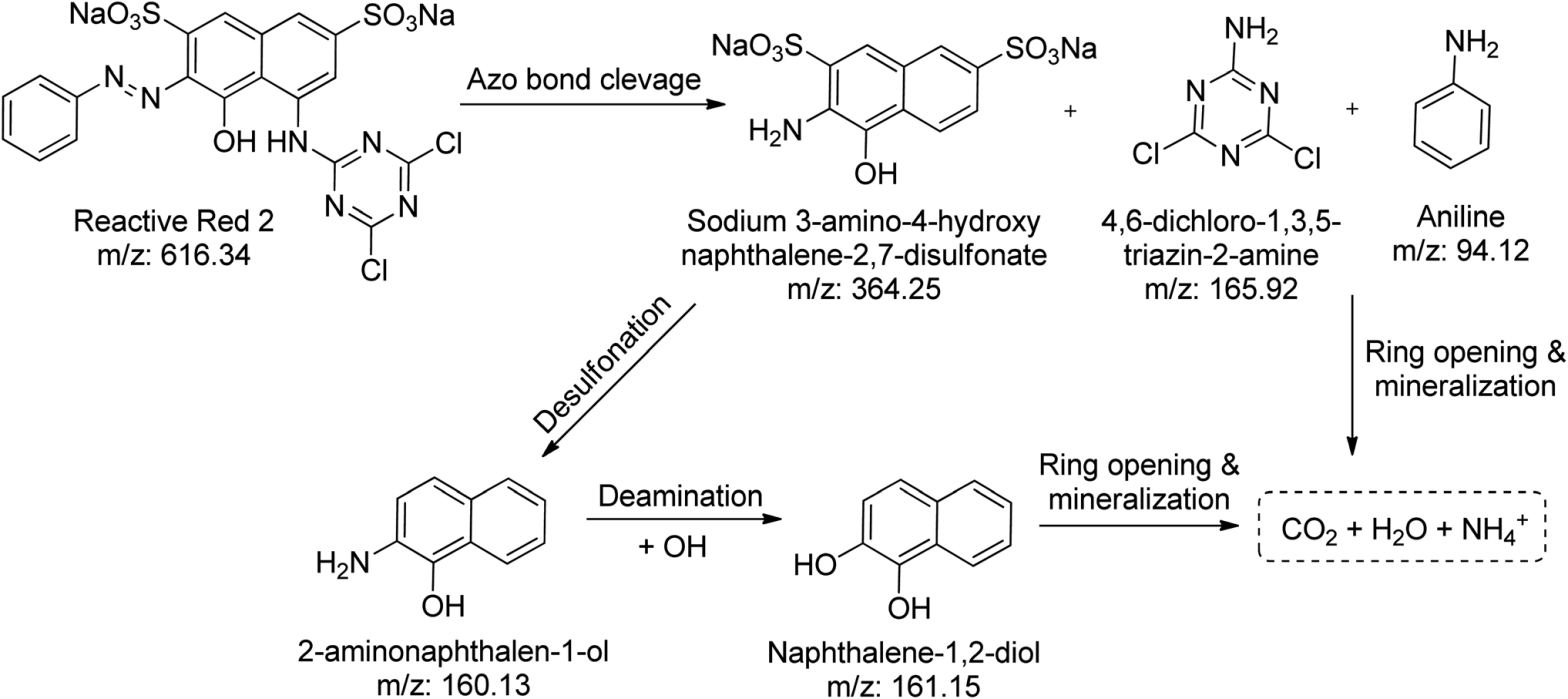
Proposed degradation pathway of RR2 in the presence of CN/GdV.

On the other hand, the azo bond between the benzene and naphthalene rings is cleaved in the RR2 dye degradation pathway (Scheme 3), resulting in the formation of three intermediates, namely sodium 3-amino-4-hydroxynaphthalene-2,7-disulfonate (*m*/*z*: 364.25), 4,6-dichloro-1,3,5-triazin-2-amine (*m*/*z*: 165.92), and aniline (*m*/*z*: 94.12). The *m*/*z* peaks of the byproducts produced during the irradiation of reactive red 2 dye were analyzed and identified using LC-MS (Fig. 7b). Further de-sulfonation of the intermediate sodium 3-amino-4-hydroxynaphthalene-2,7-disulfonate yields 2-aminonaphthalen-1-ol (*m*/*z*: 160.13), which when deaminated yields naphthalene-1,2-diol (*m*/*z*: 161.15). The intermediates, namely naphthalene-1,2-diol, 4,6-dichloro-1,3,5-triazin-2-amine, and aniline, may finally go through a ring-opening process and then mineralize into low molecular weight compounds.

### 3.4 Photocatalytic mechanism

**Fig. 8:**
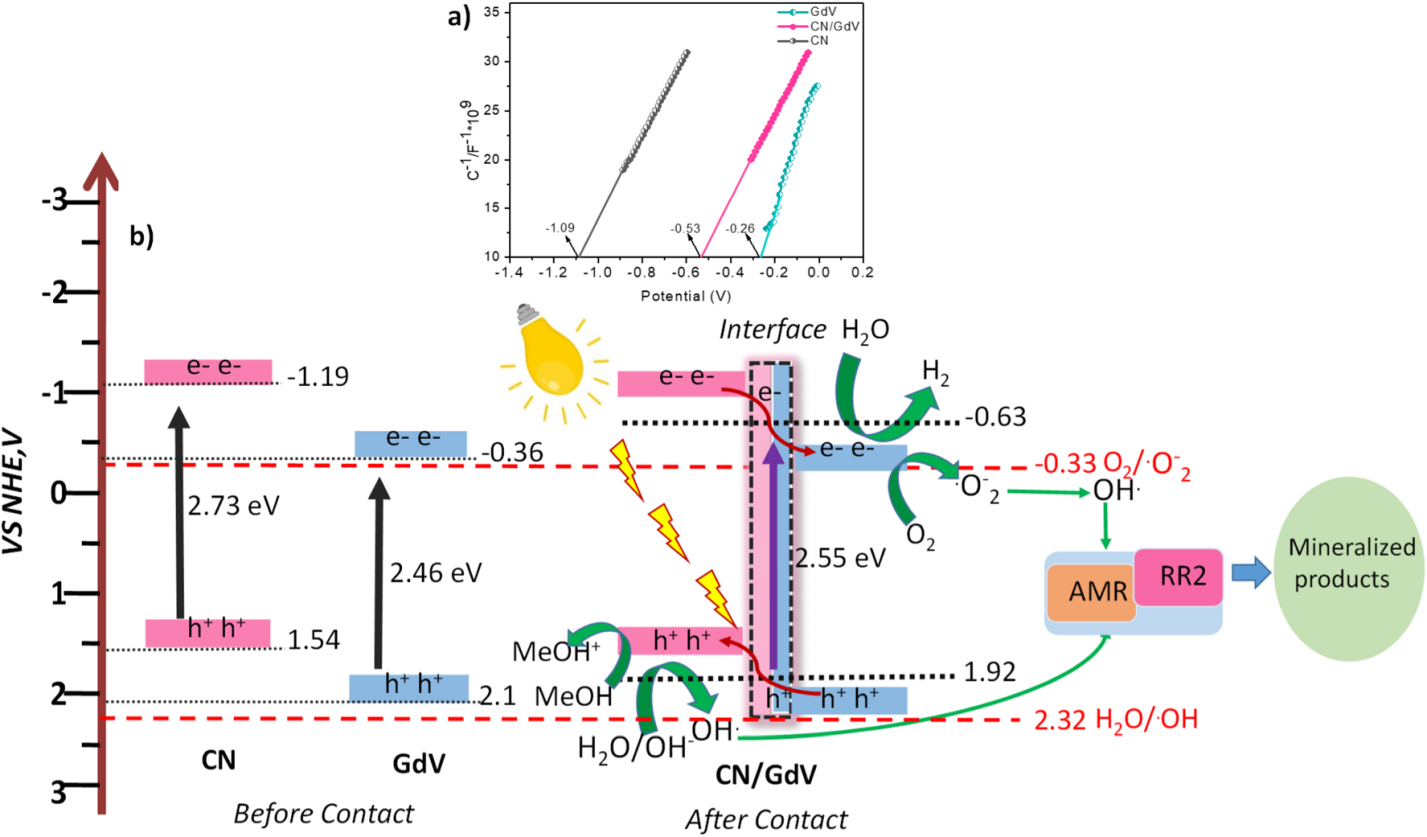
(a) Mott–Schottky plot. (b) Proposed mechanism for the photocatalysis.

Based on the findings of the UV-DRS, scavenger studies, and Mott–Schottky plots, a plausible mechanism for photocatalysis was discussed. Fig. 8a provides the Mott–Schottky plots for the synthesized materials. All three materials displayed an n-type semiconductor nature and hence the potentials of CN, GdV, and CN/GdV were found to be −1.09, −0.26, and −0.53 V, respectively. As a result, the observed Mott–Schottky results were considered to be +0.1 to the conduction band potential (*E*_CB_). It is simple to locate the valence band potential (*E*_VB_) for various materials based on the bandgap and the resulting *E*_CB_. The potential mechanism of the CN/GdV heterostructure for photocatalytic hydrogen evolution and dye degradation is shown in Fig. 8b. Since CN and GdV both absorb visible light, the development of the heterostructure happens after contact. When light has been irradiated, the electrons in CN's VB excite to its CB and exhibit a propensity to migrate to the CB of GdV over the interface, resulting in the formation of a type-II heterostructure. These electrons are used to reduce water and evolve hydrogen. In dye degradation, electrons are utilized to reduce dissolved oxygen and form superoxide radical anions, later forming hydroxyl radicals in the presence of water. The oxidation of the sacrificial agent and the formation of hydroxyl radicals are both aided by the holes in the VB of CN. According to Schemes 2 and 3, the hydroxyl radicals generated on both sides interact with dyes and mineralize to produce eco-friendly products. The reduced charge-carrier recombination during photoredox reactions may be responsible for the increased photocatalytic activity in hydrogen evolution and dye degradation in the type-II CN/GdV heterostructure.

### 3.5 Regeneration studies

**Fig. 9:**
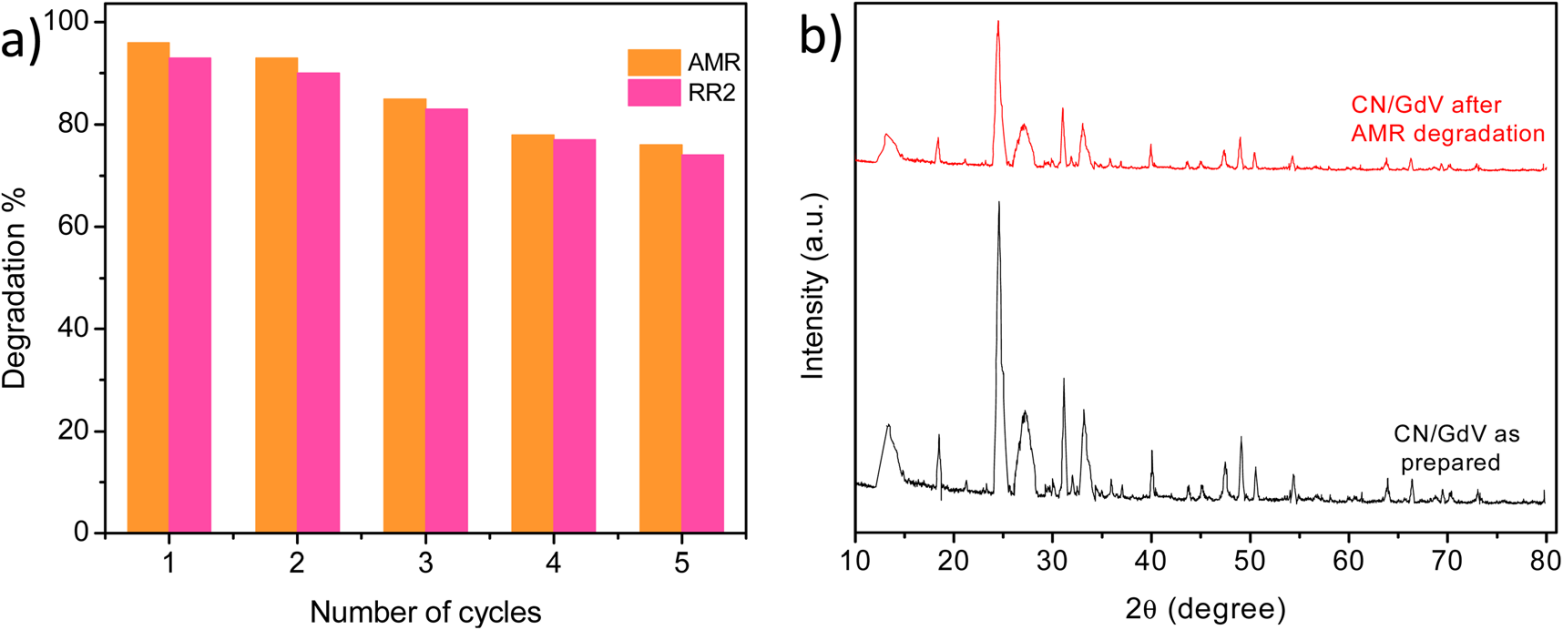
(a) Regeneration studies. (b) XRD of the CN/GdV heterostructure.

For a catalyst's practical applications, stability and reusability are crucial qualities. As a result, the stability study of CN/GdV was evaluated, and the findings in Fig. 9a show that even after 5 cycles, CN/GdV could still degrade 76% and 74% of AMR and RR2, respectively. The structural analysis of the catalyst in use was also assessed using XRD. Fig. 9b displays the XRD of CN/GdV before and after it was used to degrade AMR. The peak positions showed little variation; however, the intensity of the peaks decreased. Fig. S3† displays the results of SEM for the CN/GdV photocatalyst before and after 5 cycles of photocatalytic dye degradation activity. Once the photocatalyst's pore structure was destroyed, it began to resemble clumps of particles that had begun to agglomerate. These findings demonstrate the high structural stability of CN/GdV.

## 4. Conclusions

An efficient hydrothermal method was developed for the decoration of GdVO_4_ pellets around g-C_3_N_4_ sheets to generate a g-C_3_N_4_/GdVO_4_ heterostructure. The ability of the materials to absorb visible light toward the water splitting reaction to produce hydrogen and to degrade harmful dyes into environmental beneficial chemicals were evaluated. Compared to pure CN and GdV the stability and efficiency were found to increase upon generating the heterostructure through the interface between GdV and CN. AMR and RR2 degradation intermediates were analyzed using LC-MS. With the aid of the bandgap and Mott–Schottky results, the mechanism for the photocatalytic reactions involved in hydrogen evolution and dye degradation were examined, and the existence of a type-II heterostructure between CN and GdV was determined. The CN/GdV heterostructure demonstrated its ability to simultaneously degrade two azo dyes with not much loss of efficiency. All of the results showed the CN/GdV heterostructure's versatility as a material that could be used to address environmental and energy-related problems.

## Conflicts of interest

There are no conflicts to declare.

## Supplementary Material

RA-013-D3RA02949B-s001
