# Supplementary material for: Efficient light-driven hydrogen evolution and azo dye degradation over the GdVO4@g-C3N4 heterostructure
Source: RSC Adv. 2023 Jul 7;13(30):20417–29. doi: 10.1039/d3ra02949b (PMC10326889; doi:10.1039/d3ra02949b)
Supplement: RA-013-D3RA02949B-s001 [file RA-013-D3RA02949B-s001.pdf]

Electronic Supplementary Material (ESI) for RSC Advances.
This journal is © The Royal Society of Chemistry 2023

Efficient Light Driven Hydrogen Evolution and Azo Dyes Degradation over $GdVO_4$@g-C_3N_4 Heterostructure

Fahad A Alharthi*, Adel El Marghany, Naaser A. Y. Abduh, Imran Hasan*

Department of Chemistry, College of Science, King Saud University, Riyadh-11451, KSA

Email: fharthi@ksu.edu.sa

Email: amarghany@ksu.edu.sa

Email: 439106262@student.ksu.edu.sa

Email: iabdulateef@ksu.edu.sa *Contact: +966-507976713*

Corresponding authors

Imran Hasan (I.H.) *Email: iabdulateef@ksu.edu.sa* *Contact: +966-507976713*

Fahad A Alharthi (F.A.A.) *Email: fharthi@ksu.edu.sa*

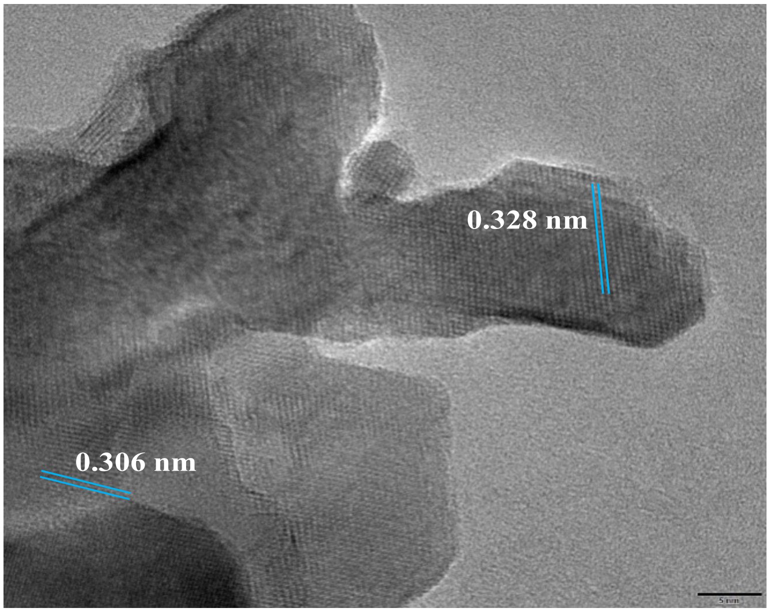

Fig. S1 HRTEM of CN/GdV heterostructure

The HR-TEM image (Fig. S1) reveals a close interface formed between two phases of CN and GdV. Consistent with the XRD, the detected lattice fringes of d = 0.328 nm, and d = 0.306 nm, correspond to the (211) crystallographic planes of GdV and (002) crystallographic plane of CN, respectively. Furthermore, this strong interaction facilitates rapid charge transfer after visible light excitation, which boosts the photocatalytic activity.

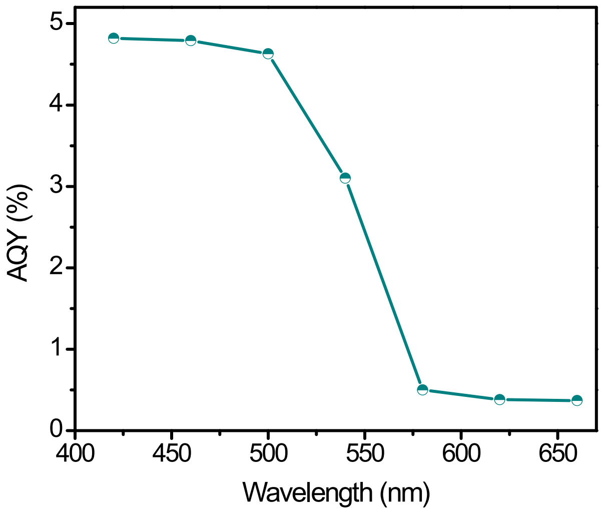

Fig S2: AQY of H_2 evolution in presence of CN/GdV

Fig. S2 shows the Apparent quantum yield (AQY) of H_2 evolution in presence of CN/GdV. AQY was determined using a 420 nm band pass filter and calculated using the formula;

AQY (%) = 2 X The number of H_2 evolved/The number of incident photons X 100

The AQY of CN/GdV is found to 4.82 % and shows good efficiency towards the absorption of light under visible region.

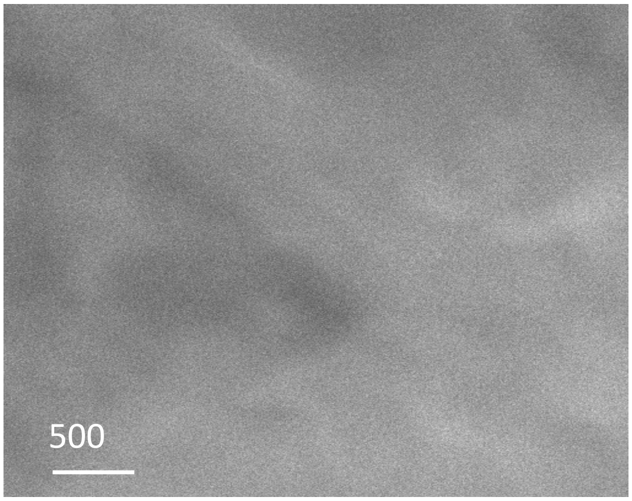

Fig. S3. SEM monograph of CN/GdV after 5 cycles of photocatalytic degradation of AMR.

Fig. S3 displays the results of SEM looking at the morphology of the CN/GdV photocatalyst before and after the 5 cycles of photocatalytic dye degradation activity. Once the photocatalyst's pore structure is destroyed, it begins to resemble clumps of particles that have begun to agglomerate. This shows the moderate stability of the CN/GdV after some cycles.
